# Gene Unprediction with Spurio: A tool to identify spurious protein sequences

**DOI:** 10.12688/f1000research.14050.1

**Published:** 2018-03-02

**Authors:** Wolfram Höps, Matt Jeffryes, Alex Bateman

**Affiliations:** 1 European Molecular Biology Laboratory, European Bioinformatics Institute (EMBL-EBI), Hinxton, CB10 1SD, UK

**Keywords:** Gene prediction, machine learning, gaussian process model, bioinformatics, sequence analysis

## Abstract

We now have access to the sequences of tens of millions of proteins. These protein sequences are essential for modern molecular biology and computational biology. The vast majority of protein sequences are derived from gene prediction tools and have no experimental supporting evidence for their translation.  Despite the increasing accuracy of gene prediction tools there likely exists a large number of spurious protein predictions in the sequence databases.  We have developed the Spurio tool to help identify spurious protein predictions in prokaryotes.  Spurio searches the query protein sequence against a prokaryotic nucleotide database using tblastn and identifies homologous sequences. The tblastn matches are used to score the query sequence’s likelihood of being a spurious protein prediction using a Gaussian process model. The most informative feature is the appearance of stop codons within the presumed translation of homologous DNA sequences. Benchmarking shows that the Spurio tool is able to distinguish spurious from true proteins. However, transposon proteins are prone to be predicted as spurious because of the frequency of degraded homologs found in the DNA sequence databases. Our initial experiments suggest that less than 1% of the proteins in the UniProtKB sequence database are likely to be spurious and that Spurio is able to identify over 60 times more spurious proteins than the AntiFam resource.

The Spurio software and source code is available under an MIT license at the following URL: https://bitbucket.org/bateman-group/spurio

## Introduction

Sequencing of genomes has now become routine with the DNA archives containing the sequences of over 100,000 complete genomes, while the direct sequencing of proteins is still low throughput and not a routine technique. Fortunately, computational methods exist to predict the protein sequence of genes from genomic DNA sequence. At least for bacterial DNA, these methods are fast and accurate. Existing tools for bacterial gene prediction claim accuracy figures of over 99% suggesting that almost all known genes in well annotated genomes are identified by these methods ^1^. However, many extra genes are predicted, some of which may be real and some of which may be false. Even if the false positive rate of the methods is only 0.1%, then within a database of 100 million proteins like UniProt we would still expect to find 100,000 spurious protein predictions. Given the widely varying quality of gene prediction pipelines still in use ^2^, we expect that the actual number of spurious proteins is likely to be much higher. An important question to address is what fraction of sequence databases are spurious gene predictions. In this paper we begin to address this problem by creating a generic tool to identify spurious proteins.

We term the task of identifying and deleting spurious gene predictions as *gene unprediction*. Gene unprediction would allow for the quality control and refinement of existing genomic annotation as well as helping to identify shortcomings in existing gene prediction pipelines. One existing tool that can aid in gene unprediction is the AntiFam database ^3^. AntiFam is a collection of profile-HMM models that can be used to identify members of potentially spurious protein families. AntiFam release 4.0 contains 65 entries that identify a range of spurious proteins. Some of these models were families initially built and included into the Pfam database (RRID:SCR_004726) ^4^, but later removed when it was pointed out they contained only spurious proteins. Many more AntiFam entries were constructed to model shadow ORFs which appear on the opposite strand of well-known genes, such as the 23S rRNA ^5^. However, the AntiFam approach does not scale well. Each family requires the effort of a curator to build it and verify its status as spurious. Many spurious proteins may be singletons, appearing only once in the sequence database and so could not form a family of spurious proteins to be included in AntiFam.

## Methods and results

Our approach to identifying spurious genes is to identify stop codons in homologous genomic DNA sequences. If we see many stop codons falling within what would be the homologous protein sequence from related organisms then we will infer that this DNA region is unlikely to be under selection at the protein level and is likely to be a spurious gene prediction. Still we must expect to find stop codons in homologous DNA sequences that are not indicative of incorrect gene prediction. Firstly the homologous DNA sequence may have sequencing errors leading to erroneous stop codons. A second reason is that stop codons are sometimes recoded for amino acids. The most prevalent examples include recoding of UGA codons as tryptophan in members of Entomoplasmatales and Mycoplasmatales ^6^, and more widely, UGA can also be interpreted as selenocysteine ^7^, as well as UAG which can be recoded as pyrrolysine in archaebacteria ^8^. Pseudogenization is a real process and so we must expect some level of stop codons to be found in homologous regions of known genes. Certain organisms have a high level of pseudogenization, in particular obligate intracellular pathogens such as buchnera species may contain up to 50% of pseudogenes ^9^.

**Figure 1.**
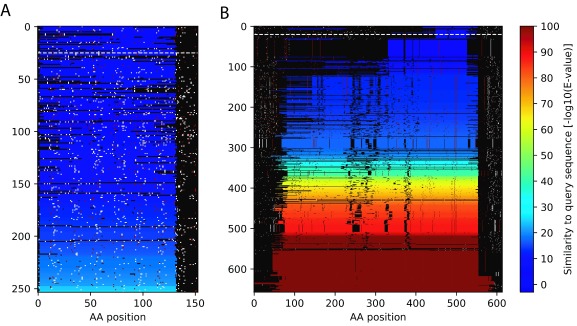
Example blizzard plots of two proteins. ( **A**) A blizzard plot of *Acinetobacter bereziniae* protein F963_00691 (UniProt accession: N8YUQ2). ( **B**) Apolipoprotein N-acyltransferase from *Mycobacterium smegmatis* (UniProt: ** A0QZ13). Each row on the plot shows the alignment region of potential protein from the tblastn search. Stop codons are shown as white pixels and methionine codons are shown as red pixels. The significance level of the match is indicated by the rainbow colour scale on the right.

Here we describe two examples that illustrate the concept of identifying spurious proteins by inspecting homologous DNA sequence. The first example is from a known spurious protein identified by the AntiFam resource. This protein is an uncharacterized protein from the microbe *Acinetobacter bereziniae* (UniProt accession: N8YUQ2) which was revealed to be a translated CRISPR YPRES repeat sequence. In Figure 1A below we show a summary visualization of the tblastn output, with each line representing a similar DNA sequence. Stop codons are identified with white pixels and give the appearance of snow falling, hence we call these blizzard plots. This is a clear case where almost every homologous DNA sequence contains stop codons throughout the alignment.

The second example ( Figure 1B) shows an example protein from UniProtKB/Swiss-Prot (Apolipoprotein N-acyltransferase from *Mycobacterium smegmatis* (UniProt: A0QZ13)). The plot is almost totally devoid of stop codons within the aligned regions. The single example stop codon is very close to the C-terminus of the protein meaning it is likely a benign change. It is interesting to see that there are black dots also within the similar sequences which represent deletions in the homologous sequence that occur in the multiple of three bases. This represents an additional line of evidence for the coding potential of the query sequence.

### Description of Spurio tool

The Spurio tool is based on running the tblastn software (RRID:SCR_011822) (we have used BLAST version 2.7.1+) using the query protein to search against a collection of microbial genome sequences. The tblastn output is parsed to include only matches more significant than the threshold E-value. We explored a range of E-values in the benchmarking and identified 10 to be a good balance between precision and recall. For the genome collection, we chose a non-redundant set of 1,507 full genomes of bacteria and archaea provided by the ENA genome database ^10^. As we mentioned earlier, Entomoplasmatales and Mycoplasmatales use an alternative genetic code, in which the UGA codon is interpreted as tryptophan ^6^. To account for this, these bacteria are processed in a separate homology search where the correct genetic code is used.

### Feature extraction and preprocessing

Our tool proceeds to transform the results of the homology search, which can be visualized as a blizzard plot, into a probability estimate for the underlying sequence to be spurious. To perform this classification, Spurio extracts three features from the set of homologous sequences. The central one, describing the relative amount of stop codons, is given in the equation F1 below. The '+1' pseudocount is a compromise for the logarithm to be applicable even if zero stop codons are found. Note also that stop codons are only counted if they fall within the region of similarity reported by tblastn. Finally, because homologous over-extension of alignments ^11^ can cause pairwise alignments to extend into non-homologous regions, we only count stop codons if they fall within the body of the tblastn matching region (Not within the first or last 10 amino acids of match positions). Additionally, Spurio uses the logarithmized number of homologous sequence hits ( Equation F2) and the protein sequence length ( Equation F3) as features, which together describe the dimensions of the corresponding blizzard plot.

$$F1\;=log\;\frac{Number\;of\;stop\;codons\;across\;all\;matched\;sequences\;+\;1}{Total\;number\;of\;amino\;acids\;in\;all\;matched\;sequences}$$

F2=log(Numberofhomologoussequences)

F3=log(Sequencelength)

### Probabilistic classification

**Figure 2.**
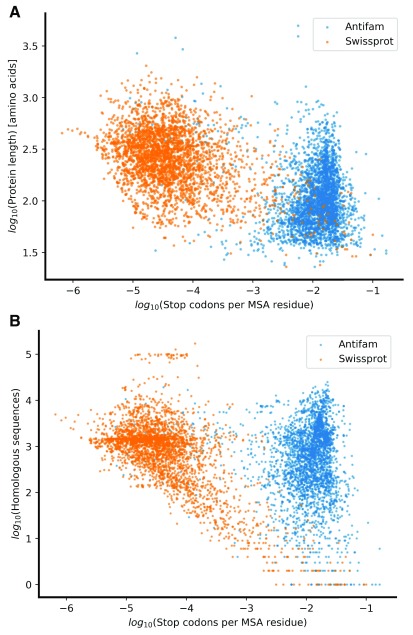
Scatter plots of the separation of AntiFam versus Swiss-Prot proteins. Protein sequences were sampled from either Swiss-Prot (3,107 sequences shown in blue) or AntiFam (3,107 spurious sequence shown in orange). After preprocessing, every protein sequence is represented by a single dot in three-dimensional space. This dataset was later used for the training and testing a probabilistic classifier. ( **A**) Shows the log length versus the normalised log of the stop codons per aligned position. ( **B**) Shows the log number of tblastn hits versus the normalised log of the stop codons per aligned position. The raw data set can be found associated with this paper.

Having extracted and preprocessed features, we use a probabilistic Gaussian process classifier ^12^ to estimate the probability of a protein to be spurious. As a supervised learning technique, the Gaussian process classifier is dependent on training samples to infer the underlying feature distribution. For this, we created a balanced sample set of protein sequences. The positive set is composed of 3,107 likely spurious proteins derived from the AntiFam resource (version 4.0) (See Supplementary File 1). The negative control set of 3,107 proteins that are genuinely translated were randomly selected from UniProtKB/Swiss-Prot (RRID:SCR_002380) (See Supplementary File 1). The distribution of these sample sequences after preprocessing suggests that the feature space is adequate for the separation of real and spurious sequences (see Figure 2).

**Figure 3.**
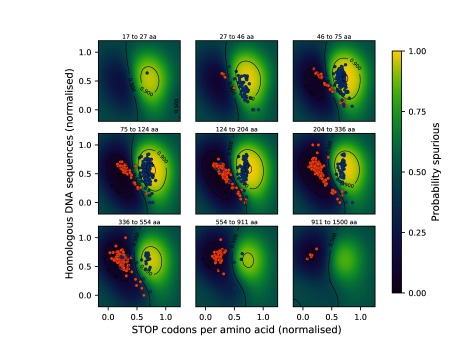
A Gaussian process classifier is used to assign probability scores to sequences, describing their likelihood to be spurious. Sequences classified as spurious are coloured blue and non-spurious proteins are coloured orange. The classification is performed in three dimensions. Shown above are cross-sections along the sequence length dimension. 500 test data samples are projected to the nearest layer in this plot. 8-fold cross validation suggests a mean prediction accuracy of 96.8%.

On this set of sample data, we trained a Gaussian process model with a radial basis function kernel implemented in the python package scikit-learn ^13^. Figure 3 shows the model after training on all samples, overlaid with 500 test samples. The performance for the whole approach is reviewed in the following section.

### Benchmarking of Spurio method

**Figure 4.**
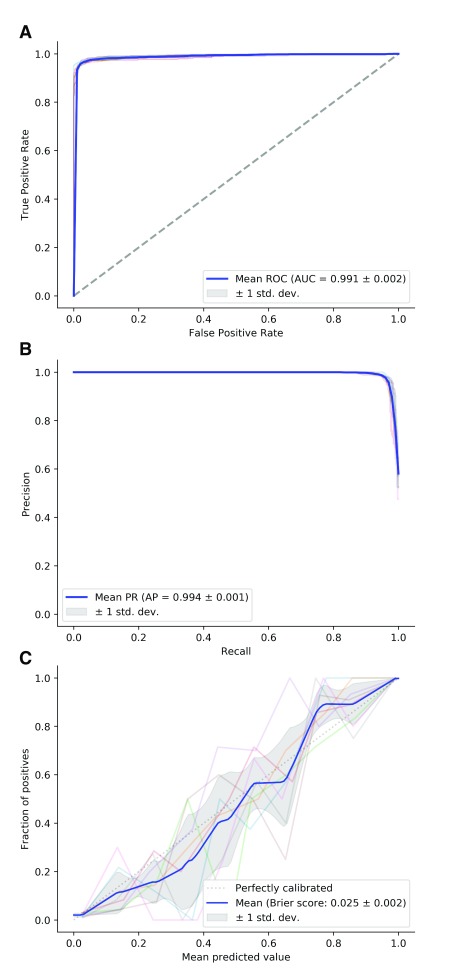
Benchmarking plots for Spurio based on 8-fold cross-validation using 5,438 training and 776 test samples per fold. The transparent lines represent the individual results of each cross-validation run, the mean over all runs is shown in blue. ( **A**) Receiver Operator Characteristic curve. ( **B**) Precision-Recall curve. ( **C**) Calibration plot showing the reliability of the model versus a perfectly calibrated model.

The Spurio software (version 1.0) was tested using 8-fold cross validation on the previously described set of 3,107 samples per class. This led to 8 iterations of 5,438 training- and 776 test samples each. Based on this procedure, we report a mean accuracy of 96.8% (training: 97.0%) and area under the curve of 0.991 (training: 0.992). The results are summarized in Figure 4.

### Practical application of the Spurio method

**Figure 5.**
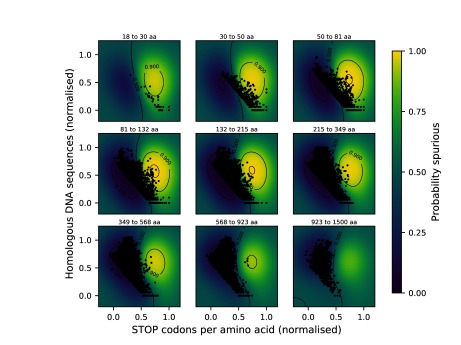
Distribution of 100,000 TrEMBL matches projected onto the Gaussian process classifier probability space. The Spurio output data can be found associated with the paper.

To further understand the performance of Spurio we ran it on 100,000 random bacterial proteins (See Supplementary File 2) from UniProtKB/TrEMBL version 2017_12 in order to estimate the number of spurious proteins (See Supplementary File 3). 5,392 Sequences did not yield any homologous sequences and were excluded. How the remaining proteins are distributed in the probability space of the Gaussian process classifier is shown in Figure 5. We see that the large majority of spurious proteins are found to be in the shorter length ranges of 30–150 amino acids as we might expect from incorrect gene predictions. As expected, we identify many more real than spurious proteins.

**Figure 6.**
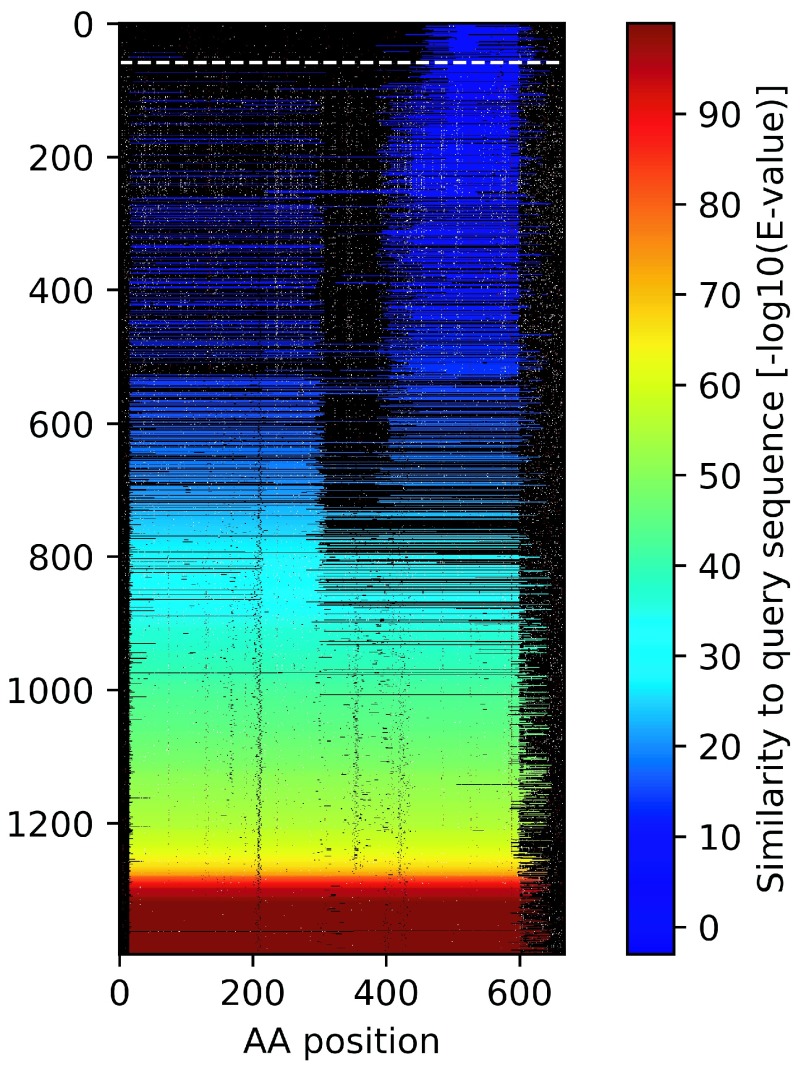
A blizzard plot of AZOBR_140218 protein from *Azospirillum brasilense* (UniProt: G8AMM6). See Figure 1 for a description of the features of the blizzard plot.

To illustrate the predictions by Spurio we have selected a representative example, the AZOBR_140218 protein from *Azospirillum brasilense* (UniProt: G8AMM6). This protein is 648 amino acids long and so would appear to be very likely a true protein coding gene. However, Spurio gives it a probability score of 0.979 indicating it is very likely to be Spurious. Inspection of the Blizzard plot ( Figure 6) shows that the DNA homologues of this sequence have a large number of stop codons. Further investigation shows that this protein is on the opposite strand to the translational GTPase TypA (UniProt: A0A060DFP7) which strongly suggests that the AZOBR_140218 protein is indeed spurious and is a shadow ORF. Interestingly searching this spurious protein for homologues identifies many proteins including some that are erroneously annotated as the enzyme 1-deoxy-D-xylulose 5-phosphate reductoisomerase (see UniProt: R5CSG3 as an example).

If we select an arbitrary threshold of 0.8 or greater to represent a spurious protein then 0.82% of the 100,000 sample of proteins are predicted to be spurious. Of these 26% have matches to Pfam which is somewhat surprising (see Table 1). However, if we consider proteins with no Pfam match we find that 3.8% of them have a Spurio score > 0.8 compared to just 0.25% of proteins with a Pfam match. Thus proteins with no Pfam match are 15 times more likely to be predicted as spurious than those with a Pfam match. If we search the sample of 100,000 proteins with AntiFam we find it identifies only 12 that are spurious (see Supplementary File 4). Therefore, Spurio is able to identify 62 times more spurious proteins than AntiFam. Of the 12 AntiFam matched proteins, 9 had Spurio scores of 0.97 or greater. The results of the AntiFam search can be found in Supplementary materials.

**Table 1.** Contingency table showing the number of matches with Spurio scores >= 0.8 versus the presence of a match to Pfam.

	No Pfam match	Pfam match
**Spurio score >= 0.8**	551	193
**Spurio score < 0.8**	13, 863	75, 638

**Figure 7.**
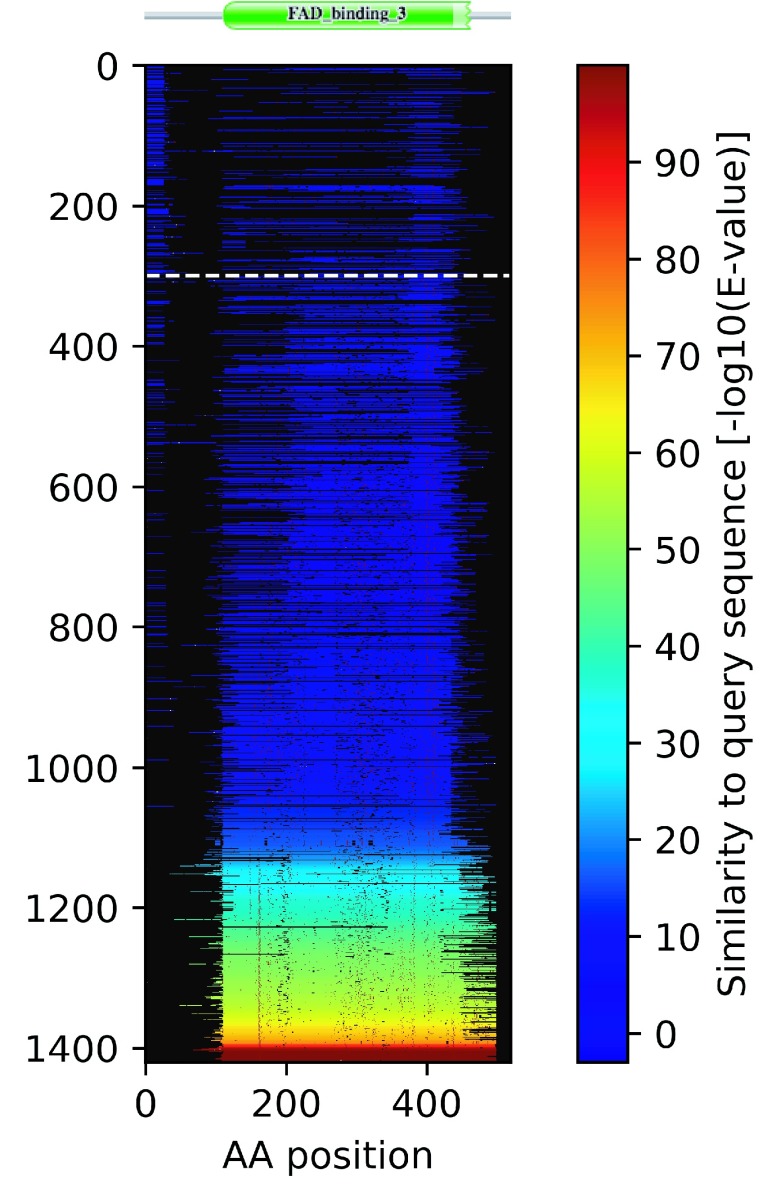
A blizzard plot of ALP79_101044 protein from *Pseudomonas savastanoi pv. fraxini* (UniProt: A0A0W8HJ99). See Figure 1 for a description of the features of the blizzard plot. The Pfam domain architecture of this protein has been added at the top of the figure.

It is interesting to highlight an example where Spurio does did not match a protein that AntiFam did. If we take the example ALP79_101044 (UniProt: A0A0W8HJ99) we find that it has a Spurio score of 0.14 and has a strong Pfam match to the FAD_binding_3 family (Pfam: PF01494). The blizzard plot ( Figure 7) shows that there is very little similarity detected to other organisms in the N-terminal 100 amino acids. It has an AntiFam match at the N-terminus of the protein from residues 1-25 to a translation of a tRNA. It seems likely that the protein should start at the methionine which is at position 31 of the existing sequence in UniProt.

**Figure 8.**
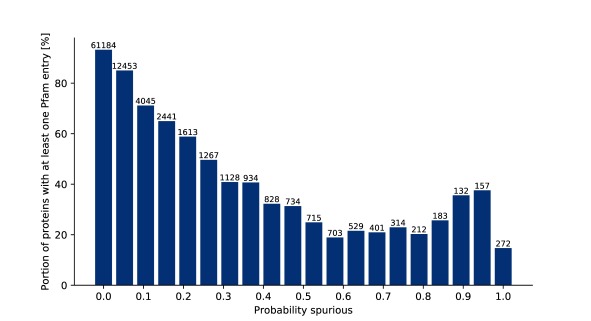
Histogram showing the proportion of sequences matching Pfam across the range of Spurio scores. 5,392 Sequences did not yield any homologous sequences and were excluded. Another 4,363 samples were processed by spurio, but were released later than the current version in InterPro and were thus excluded. The plot shows the remaining 90,245 sequences.

We continued to investigate whether sequences predicted as spurious are less likely to be members of existing protein families in Pfam than those sequences predicted to be true proteins. We would expect that spurious proteins would be unlikely to fall into Pfam families and so in a perfect world we would see the expected number of Pfam matches at a Spurio score of 0 and see no Pfam matches at a Spurio score approaching 1. Figure 8 shows that in the 100,000 sequences from TrEMBL this is the case for predicted values from zero up to 0.6. But above that value we see an excess of matches to Pfam. To understand what is causing this excess of matches to families we created a list of the top ten most frequently occurring Pfam families, shown in Table 2. Inspection shows that eight out of the top ten Pfam families are related to transposon function. It is known that there can be many copies of degraded transposons within a genome. The larger than normal number of these degraded copies compared to proteins with normal cellular functions makes them appear to be spurious proteins.

**Table 2.** Table showing the ten most prevalent Pfam families among proteins with Spurio scores >= 0.8. Pfam accessions for the families that are likely to be transposon associated are underlined.

Pfam accession / Pfam identifier	Number of matches	Pfam description
PF13610 / DDE_Tnp_IS240	14	Transposase DDE domain
PF00313 / CSD	10	Cold-shock domain
PF01609 / DDE_Tnp_1	9	Transposase DDE domain
PF03400 / DDE_Tnp_IS1	8	IS1 Transposase
PF14104 / DUF4277	8	Domain of unknown function (DUF4277)
PF13340 / DUF4096	8	Putative transposase
PF13586 / DDE_Tnp_1_2	7	Transposase DDE domain
PF00936 / BMC	6	Bacterial Microcompartment domain
PF00239 / Resolvase	6	Resolvase, N-terminal domain
PF13358 / DDE_3	5	DDE superfamily endonuclease

**Figure 9.**
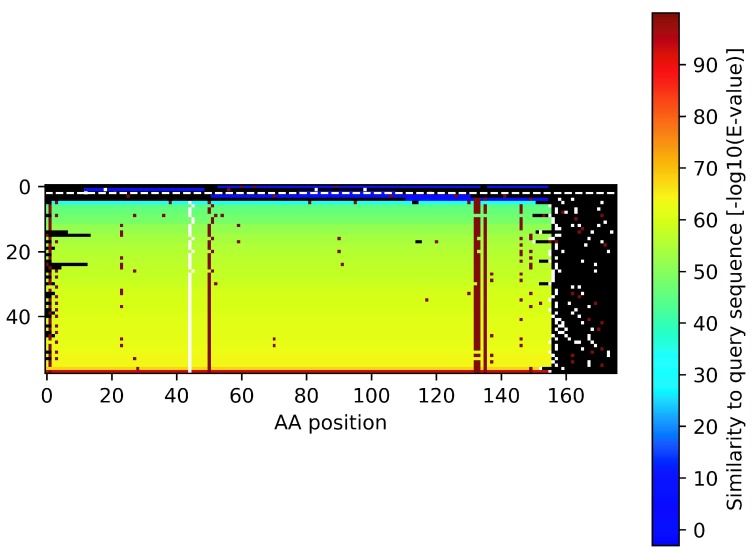
A blizzard plot of the selenoprotein GrdA from *Carboxydothermus hydrogenoformans* (UniProt: Q3A9J5). See Figure 1 for a description of the features of the blizzard plot.

We expected that selenoproteins may present problems for the Spurio method. To examine this we took an example selenoprotein GrdA from *Carboxydothermus hydrogenoformans* (UniProt: Q3A9J5) and ran Spurio on it. We found that indeed it was scored as 0.891 probability to be spurious (see Figure 9). One can clearly see in the blizzard plot the conserved selenocysteine position as a column of stop codons. It is interesting to note that selenoproteins that have been mispredicted to contain premature stop codons are unlikely to be predicted as spurious.

## Discussion

The identification of spurious genes is an area of genomic annotation that has received very little attention. This is partly due to the difficulty of proving that a gene is not expressed in any condition. We have made a generic tool to discover spuriously predicted proteins from bacterial genome sequences. Our attempt is reasonably successful, but we find that while we can indicate likely spurious genes, there are some failure modes that mean that the Spurio results should be considered indicative and that they will require inspection for some applications. For example, transposon related genes are apt to be predicted as spurious because they have many pseudogenized homologues. It may be possible that this could be turned into a positive attribute to help identify regions of a genome with high predicted spuriousity that may be transposons.

In order to improve the accuracy of Spurio we recommend that users focus on proteins that do not fall into known Pfam families as well as short proteins less than 150 amino acids in length. A use case where Spurio may be particularly appropriate is in the case of overlapping genes. If genes are called on opposite strands then Spurio could be used to detect if either or both the genes may be due to spurious gene prediction. A preliminary study of 21,452 genes in overlapping pairs (>50 nucleotide overlap) showed that 8.7% (1,867) of them had a Spurio score of 0.8 or higher (See Supplementary File 5).

Spurio could be further developed by the addition of new features for training the model. Possible features could include the fraction of residues covered by Pfam domains. We would expect that spuriousness would negatively correlate with this feature. Also the number or proportion of insertions or deletions may carry useful information to discriminate real from spurious genes. It is worth noting that Pearson showed that protein sequences are essentially random and so features based on protein sequence or composition may not be informative ^14^. Because we have found that transposons have a propensity to be predicted as spurious it may be beneficial to have a feature that measures how many times a protein matches within a particular genome, i.e. the average copy number. Transposons are often found in multiple copies per genome. We might expect this to be higher for transposon proteins.

Although we did not see amino acid recoding to be an important factor in testing Spurio, it would be possible to attempt to make an ab initio prediction of recoding of stop codons. For example if we saw a TGA stop codon was consistently aligned to cysteine residues in the tblastn output we could predict that stop codon as a selenocysteine position. This may make an incremental enhancement of prediction accuracy.

With a method to assess the level of spurious proteins in hand we can assess the quality of a variety of protein sequence datasets. One future avenue to explore, would be to use Spurio as a quality control metric for complete proteomes. By looking at the fraction of predicted spurious proteins on a per proteome basis one could assign a quality index. In addition, we could also investigate how the quality of protein datasets has changed over time. It has been suggested that the quality of databases and their annotations may degenerate over time due to new protein sequences being based on previous erroneous protein sequences. Spurio gives us an initial estimate of 0.82% of TrEMBL proteins being spurious. Depending on your perspective this might be considered reassuringly low, or alarmingly high. Whatever your perspective, we believe that Spurio gives us a new and important tool to address issues of gene misprediction and we hope this will motivate further work in the area of gene unprediction.

## Operation

To run *Spurio*, *blast* ^15^ and *bedtools* ^16^ must first be installed. *Spurio* has several Python dependencies, which are listed in the *requirements.txt* file. *Spurio* requires Python 3.

## Software availability

Spurio software and source code is available at: https://bitbucket.org/bateman-group/spurio

Archived source code as at time of publication: https://doi.org/10.5281/zenodo.1184437 ^17^

License: MIT

## Supplementary material

**Supplementary File 1: Training data for the Spurio classifier.** This file contains the 3,107 positive AntiFam proteins and the negative set of 3,107 UniProtKB/Swiss-Prot proteins. As well as the UniProtKB identifier we include the feature values used by the classifier.

**Supplementary File 2: List of the 100,000 protein sample from UniProtKB/TrEMBL.** List accession numbers of the 100,000 bacterial protein sample randomly taken from UniProtKB/TrEMBL from UniProtKB version 2017_12.

**Supplementary File 3: Spurio matches to the 100,000 sample of UniProtKB/TrEMBL.** This file contains the Spurio match data for the 94,602 proteins which could be scored by Spurio from the 100,000 random TrEMBL sample sequences. Column 1 contains the TrEMBL accession, column 2 contains the Spurio score, Columns 3 to 5 contain the feature values used by the Spurio classifier.

**Supplementary File 4: AntiFam matches to the 100,000 sample of UniProtKB/TrEMBL.** This tab delimited file describes the 12 AntiFam matches found in the 100,000 random TrEMBL sample sequences. The Spurio scores for each are included for reference.

**Supplementary File 5: Spurio matches to the 21,452 overlapping proteins from UniProtKB/TrEMBL.** This file contains the Spurio match data for the 21,452 overlapping proteins sampled from UniProtKB/TrEMBL. Column 1 contains the TrEMBL accession, column 2 contains the Spurio score, Columns 3 to 5 contain the feature values used by the Spurio classifier.
