# Supplementary material for: Gene Unprediction with Spurio: A tool to identify spurious protein sequences
Source: F1000Res. 2018 Mar 2;7:261. [Version 1] doi: 10.12688/f1000research.14050.1 (PMC5897793; doi:10.12688/f1000research.14050.1)
Supplement: Peer review history

# Peer review: Referee response for version 1

**DOI:** 10.5256/f1000research.15280.r31445

**Published:** 2018-04-12

**Authors:** Daniel H. Haft

**Affiliations:** 1 National Center for Biotechnology Information (NCBI), National Library of Medicine (NLM), National Institutes of Health (NIH), Bethesda, MD, USA

Prokaryotic structural and functional annotation improves over time as growing resources, such as Pfam or CDD, add to the collections of rules that automated annotation pipelines can call on for genome analysis.  A considerable amount of genomic “dark matter” remains in the form of proteins not currently reached by any annotation rule.   Most large clusters in the dark matter really do represent real proteins in need of characterization and a name.  But some merely appear to be real, and to be suitable for the invention of new “domain of unknown function” protein families, when actually they reflect a long legacy of false-positive errors in the prediction of protein-coding regions.  The authors here introduce Spurio, a tool that finds suspicious proteins whose would-be homologs from related DNA show a statistically damning “blizzard” of stop codons spread across their sequence alignments.

 As the authors make clear, Spurio does not provide a clear yes/no decision for which proteins are real.  It provides merely a list of proteins that is highly enriched in false predictions, vs. those lacking evidence of falseness.  Some protein families, encoded by selfish genetic elements such as transposons, have members decay into pseudogenes so frequently a blizzard of stop codons can mislead.  What Spurio actually offers is a new analytical metric that can integrate into workflows for building new protein families, or for deprecating old ones, or for culling bad data from large databases such as UniProt and RefSeq.   Some human review, or use in combination with other indicators, may be necessary for most uses.

 Spurio is likely to find its most enthusiastic users among the biocurators and bioinformaticians who build new protein family definitions such as the HMMs of Pfam, and the developers of prokaryotic annotation pipelines such as RAST or PGAP.   Because so many researchers in the biology and biochemistry of bacteria and archaea depend on these resources, as they try to make better sense of genomic and metagenomic “dark matter,” Spurio may contribute positively to the infrastructure of bioinformatics, with most beneficiaries unaware of its theory and its role.

I have read this submission. I believe that I have an appropriate level of expertise to confirm that it is of an acceptable scientific standard.

# Peer review: Referee response for version 1

**DOI:** 10.5256/f1000research.15280.r31447

**Published:** 2018-03-13

**Authors:** Arne Elofsson

**Affiliations:** 1 Science for Life Laboratory, Stockholm University, Solna, Sweden

The authors report a novel tool to detect "spurious" hits in gene assignments. The methodology is based on the assumption that "homologous gene sequences" that  contain a substantial amount of stop codons indicate that a gene is "not under selective pressure. 

 The tool seems to be superior to the earlier tool (antifam) and is therefore a useful tool for automatic  annotations of genomes.

 Although this assumption most likely is correct in most cases - it also might miss non-spurious genes, in particular orphan genes. It is generally accepted that there exist a birth and death process where novel (orphan) genes can occur from non-coding regions and also that existing genes turns into pseudo-genes. This could be discussed further.

 There exist quite good set of orphans in drosophila and yeast - it would be interesting to see how spurio would rank these. (I am aware that this tool is mainly for bacteria - but at least in yeast most of these orphan genes are single exon so it should be possible to run it I think).

I have read this submission. I believe that I have an appropriate level of expertise to confirm that it is of an acceptable scientific standard.
